# PDCD10 promotes the aggressive behaviors of pituitary adenomas by up-regulating CXCR2 and activating downstream AKT/ERK signaling

**DOI:** 10.18632/aging.204206

**Published:** 2022-08-11

**Authors:** Jingdian Liu, Junwen Wang, Weidong Tian, Yu Xu, Ran Li, Kai Zhao, Chao You, Yuan Zhu, Joerg Walter Bartsch, Hongquan Niu, Huaqiu Zhang, Kai Shu, Ting Lei

**Affiliations:** 1 Department of Neurosurgery, Tongji Hospital Affiliated to Tongji Medical College, Huazhong University of Science and Technology, Wuhan, China; 2 Department of Neurosurgery, The First Affiliated Hospital, School of Medicine, Shihezi University, Shihezi, China; 3 Department of Neurosurgery, University of Duisburg-Essen, Essen, Germany; 4 Department of Neurosurgery, University Marburg, Marburg, Germany

**Keywords:** PDCD10, pituitary adenomas, proliferation, migration, invasion

## Abstract

As the second most common primary intracranial neoplasms, about 40% of pituitary adenomas (PAs) exhibit aggressive behaviors and resulting in poor patient prognosis. The molecular mechanisms underlying the aggressive behaviors of PAs are not yet fully understood. Biochemical studies have reported that programmed cell death 10 (PDCD10) is a component of the striatin-interacting phosphatase and kinase (STRIPAK) complex and plays a dual role in cancers in a tissue- or disease-specific manner. In the present study, we report for the first time that the role of PDCD10 in PAs. Cell proliferation, migration and invasion were either enhanced by overexpressing or inhibited by silencing *PDCD10* in PA cells. Moreover, PDCD10 significantly promoted epithelial–mesenchymal transition (EMT) of pituitary adenoma cells. Mechanistically, we showed that the expression of CXCR2, together with phosphorylation levels of AKT and ERK1/2 were regulated by PDCD10. Activation of CXCR2 inversed inactivation of AKT/ERK signal pathways and the tumor-suppressive effects induced by *PDCD10* silencing. Finally, the pro-oncogenic effect of PDCD10 was confirmed by *in vivo* tumor grafting. Taken together, we demonstrate for the first time that PDCD10 can induce aggressive behaviors of PAs by promoting cellular proliferation, migration, invasion and EMT through CXCR2-AKT/ERK signaling axis.

## INTRODUCTION

Arising in the adenohypophysis, pituitary adenomas (PAs) are the second most common primary intracranial neoplasms and account for nearly 25% of central nervous system tumors in adults [1]. Although the majority of PAs are benign, 40% of PAs exhibit aggressive behaviors such as locoregional invasiveness, rapid growth, and a high tendency to recur, which represented a poor patient prognosis [2, 3]. At present, chemotherapy and surgical resection are major modalities for PA treatment [4]. However, aggressive PAs can develop resistance to these treatment modalities. It is reported that the rate of recurrence of PAs after resection is high with about 7~58% [5, 6]. Besides, the therapeutic effects of chemotherapy are not satisfactory, due to the strong cytotoxic effects of common anti-tumor drugs [7, 8]. Therefore, deep exploration of the pathogenesis of tumor invasion is desperately needed to identify new molecular targets and drugs for aggressive PAs.

**Figure 1:**
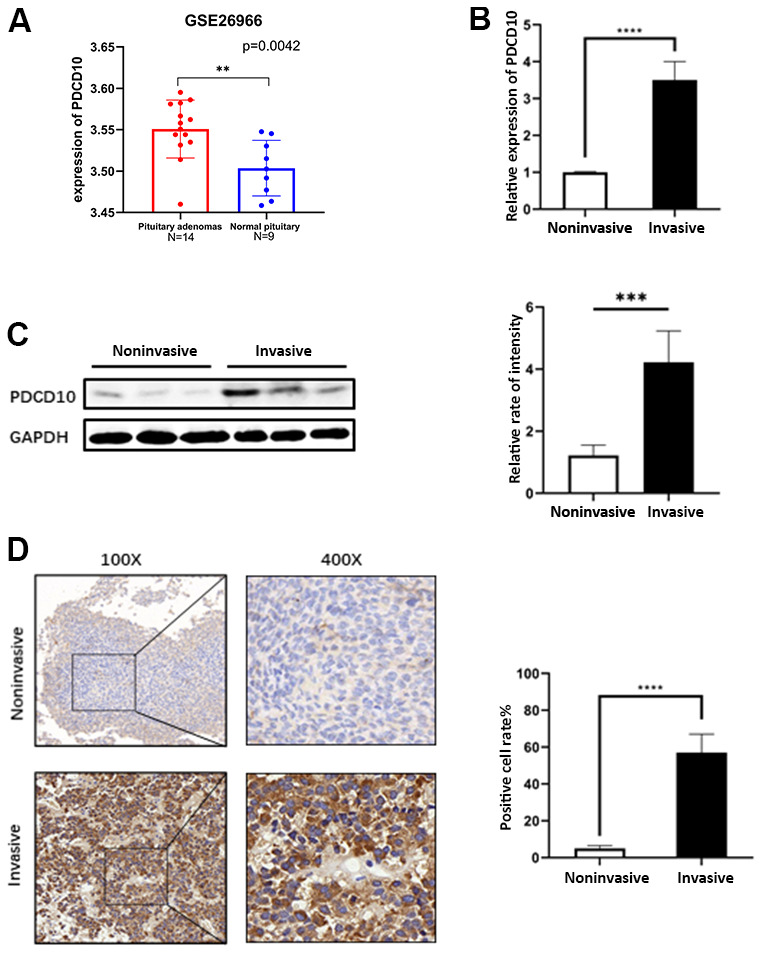
**Expression of PDCD10 in human pituitary adenomas.** (**A**) A dataset (GSE26966) was obtained from the GEO database to compare the mRNA expression level of PDCD10 between PA tumor tissues (N=14) and normal pituitary tissue (N=9). (**B**) Relative mRNA expression levels of *PDCD10* by RT-qPCR in invasive (n=15) and non-invasive (n=15) pituitary adenomas. Non-invasive values were set to 1. (**C**) Representative Western blots showed the expression level of PCDC10 protein (25 kDa) in invasive and noninvasive pituitary adenomas. Band intensities were quantified and normalized to GAPDH. (**D**) Representative images of immunohistochemistry evaluated the expression of PDCD10 in invasive and non-invasive (n=15) pituitary adenomas. **P < 0.01; ***P < 0.001; ****P < 0.0001.

Programmed cell death 10 (PDCD10), also termed “cerebral cavernous malformations 3” (CCM3) or “TF-1 cell apoptosis-related gene 15” (TFAR15), is widely expressed in various types of human cells [9]. Originally, PDCD10 was described as an apoptosis-related factor that is up-regulated upon apoptotic stimuli and inhibits natural cell death of HEK293 cells and fibroblast cells [10, 11]. Besides, PDCD10 was found to participate in the formation of a STRIPAK complex and regulates a wide variety of cellular processes [12]. For example, PDCD10 can recruit germinal centre kinase III (GCKIII) kinase to striatins (STRNs) to regulate cell proliferation and migration [13, 14]. As one of the CCM proteins family, PDCD10 also plays a critical role in angiogenesis in the central nervous system. Similar to CCM1 and CCM2, mutations in the *PDCD10* gene were responsible for cerebral cavernous malformations which are characterized by aberrant angiogenesis in the brain [15, 16]. In recent years, the function of PDCD10 in tumors has been increasingly emphasized. Several studies reported that expression of PDCD10 was altered and associated with tumor progression in various types of cancers including ovarian cancer, breast cancer, hepatocellular carcinoma, and non-small cell lung cancer, while a study on glioblastoma indicated a dual role and disease-specificity of the function of PDCD10 [17–21]. Given these multiple functions in tumors, PCDC10 could be involved in PA pathology. However, the function of PDCD10 in PAs has not yet been described. A dataset from the GEO database shows that the expression level of PDCD10 was increased in PAs tissues compared with normal counterparts (Figure 1A), which suggests a pro-oncogenic effect of PDCD10 in PAs.

As a G-protein-coupled receptor, CXC motif chemokine receptor 2(CXCR2) is predominantly expressed on the surface of inflammatory cells including neutrophils, oligodendrocytes, mast cells, eosinophils, and monocytes [22]. However, CXCR2 has also been found on epithelial cells, endothelial cells, some neuroendocrine cells (e.g., pituitary), and various tumor cells [23, 24]. We previously showed that PDCD10 promotes tumor progression *in vivo* by enhancing CXCL2-CXCR2 signaling in glioblastoma [25]. In this study, we systematically explored the functional role of PDCD10 in PAs and demonstrated that PDCD10 could regulate activation of AKT/ERK signaling pathways by altering the protein expression level of CXCR2 to modulate cellular proliferation, migration, invasion and EMT in PAs.

## MATERIALS AND METHODS

### Human pituitary adenoma samples

From July 2017 to October 2018, 15 invasive and 15 noninvasive PA samples were collected from patients who underwent surgery at the Department of Neurosurgery, Tongji Hospital affiliated with Tongji Medical College, Huazhong University of Science and Technology. Knosp grading scheme and intraoperative findings were employed to determine the invasiveness of the tumor [26]. The clinical characteristics of the patients were summarized in Table 1. The median age of patients at surgery was 43.5 years (range 23-68 years). Thirteen were male (43.3%), and 17 were female (56.7%) with a male to female ratio of 1:1.31. Tumor samples were used for immunohistochemical (IHC) staining, protein, and RNA extraction. The positive cell rate was quantified using ImageJ software (NIH).

**Table 1:** The clinical characteristics of the patients.

	**Invasive PA**	**Noninvasive PA**
**Age**		
Mean	41.7	44.3
Range	23-68	28-65
**Gender**		
Male	8	6
Female	7	9
**Knosp Grade**		
I	0	7
II	0	8
III	5	0
IV	10	0

### Cell culture and transfection

Att-20 cell line was established from mouse ACTH-secreting pituitary tumors by Buonassisi et al. [27], and TtT/GF is a murine folliculo-stellate-like cell line deriving from a thyrotrophic pituitary tumor [28]. The two mouse PA cell lines were cultured in Dulbecco’s modified Eagle’s medium (DMEM, Gibco, Carlsbad, USA) containing 10% fetal bovine serum (FBS, Gibco) and 1% Penicillin/Streptomycin and were placed in a thermostatic incubator at 37° C with 5% CO^2^. Recombinant mouse CXCL2 protein (R&D System) was added into the culture medium to study the signaling pathways in a subset of experiments as indicated.

Lentiviral shRNA vector for mouse PDCD10 (LV-PDCD10 71721) and control vector (Scramble shRNA, LVCON054) were purchased from GenePharma, Shanghai, China. Cells were seeded into 6-well plates to achieve 20-30% confluence before infection. And then 1 ml of serum-free medium containing lentivirus (1×10^9^ TU/ml) was added to each well. After 72h, a culture medium containing 1 mg/ml puromycin was used for 3 weeks to select PA cells with stable knockdown of PDCD10. CDS-sequence of mouse PDCD10 was inserted into pcDNA-3.1 plasmids to gain PDCD10-overexpression vectors. Cells were transiently transfected with the pc3.1-PDCD10 plasmids and control plasmids (pc3.1) by using lipo3000 (Invitrogen) according to the manufacturer’s instructions. The knockdown or overexpression of PDCD10 was confirmed by RT^2^-PCR and western blotting.

### Protein extraction and Western blotting

The protocols of cell protein extraction and western blotting were performed as previously [29]. Antibodies to PDCD10, E-cadherin, N-cadherin and Vimentin (VIM) were purchased from Proteintech Group. Antibody to GAPDH was purchased from Servicebio. Primary antibodies to CXCR2 and ki-67, and secondary antibodies for western blotting were purchased from Abcam. Antibodies to ERK1/2, p-ERK1/2 (phosphorylation site Thr202/Tyr204), STAT3, p-STAT3 (phosphorylation site Tyr705), AKT, and p-AKT (phosphorylation site Ser473) were obtained from Cell Signaling Technology, Inc.

### Cell proliferation, migration, and invasion assay

4.0×10^3^ cells were seeded into 96-well-plate in quintuplicate. After 72 hours of incubation, the absorbance was measured at 450 nm by adding a CCK8 reagent (Boster Biological Technology) to detect cell proliferation. Cell migration was evaluated by scratch assays as described previously [25]. After 16 hours of incubation, the migrated area was photographed and calculated by ImageJ software. For invasion assay, 8 mm pore transwell inserts (Corning Life Sciences) were coated with 75μl Matrigel matrix (Corning, 200 μg/mL) as upper chambers. 700μl DMEM medium with 20% FBS was added into the 24-well-plate. Meanwhile, 5.0×104 cells were suspended in a serum-free medium and were added to the inserts which were placed in a 24-well-plate. After 48h, the cells were fixed with 10% formalin and stained with 0.1% crystal violet solution. The numbers of invaded cells were counted at 200x magnification.

### Xenograft experiments

Nude mice (blab/c-nu; SJA Laboratory Animal Co. Ltd, Hunan, China) were subcutaneously injected with 5 × 10^6^ cells to establish the Att-20 and TtT/GF cell xenograft model. Tumor growth was monitored regularly and 20 days after injection, tumor sizes and weights were measured. For IHC staining, tumor samples were paraffin-embedded following 4% paraformaldehyde fixation. The IHC results were analyzed by ImageJ. For western blotting, the total protein of the tumor samples was extracted.

### Statistical analysis

Statistical software GraphPad Prism Ver.8.0 was employed for performing Statistical analysis and graphing. The student’s t-test and ANOVA were appropriately used for statistical analyses. Statistical significance was established as p<0.05.

## RESULTS

### Expression of PDCD10 in human pituitary adenomas

A dataset (GSE26966) from the GEO database shows that the mRNA expression level of PDCD10 was increased in PAs tissues compared with normal counterparts (Figure 1A). Furthermore, the expression of PDCD10 was examined between non-invasive and invasive PA tumor samples which were collected from the Neurosurgery department at Tongji Hospital. In contrast to non-invasive PAs, the mRNA and protein expression levels of PDCD10 were significantly upregulated in invasive PAs on the mRNA (Figure 1B), protein level (Figure 1C), and following IHC staining with anti-PDCD10 (Figure 1D).

### PDCD10 silencing suppresses the proliferation, migration, invasion and EMT of PA cells

**Figure 2:**
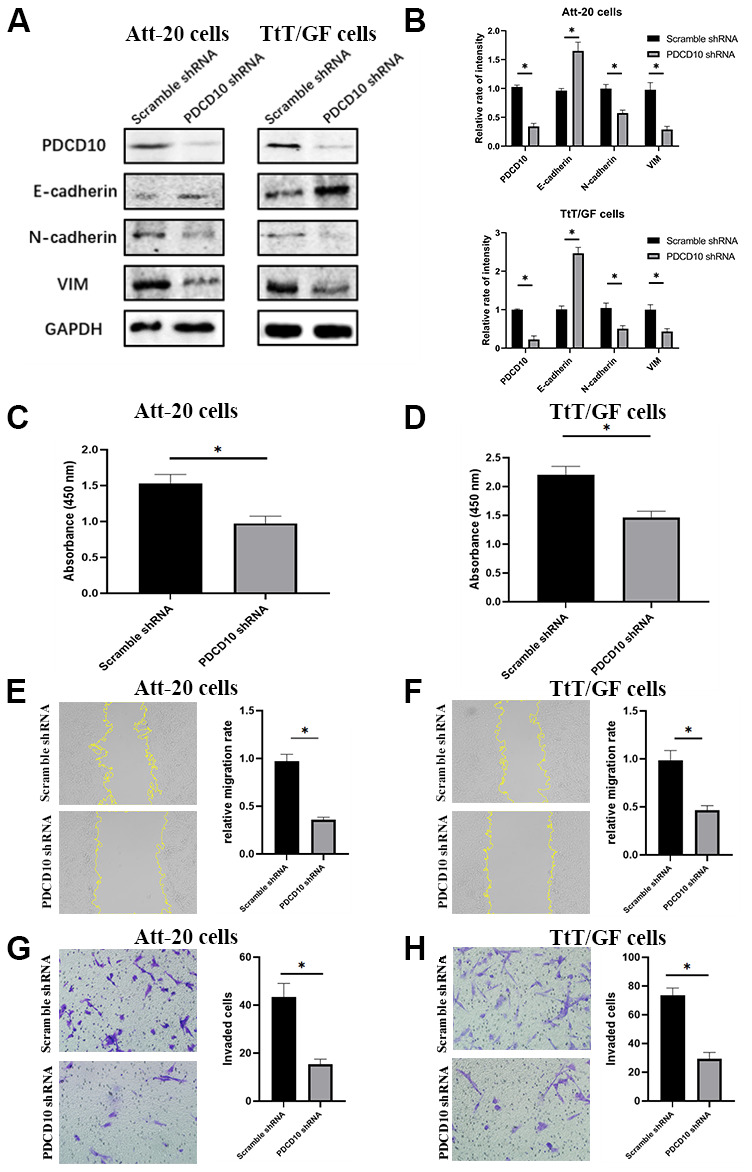
***PDCD10* silencing suppresses the proliferation, migration, invasion and EMT of PA cells.** (**A**) Western blotting was performed to detect the impact of PDCD10 silencing on the expression of EMT markers in Att-20 cells and TtT/GF cells. (**B**) Band intensities were quantified and normalized to GAPDH. (**C**, **D**) CCK-8 assay was used to assess cell proliferation capacity after *PDCD10* silencing in Att-20 and TtT/GF cells. (**E**, **F**) Scratch assay was used to examine the relative migration rates of Att-20 and TtT/GF cells (magnification:100x). (**G**, **H**) Transwell invasion assay was used to analyze the invasion potential of Att-20 and TtT/GF cells (magnification: 200x). * P < 0.05.

To explore the functional role of PDCD10 in PAs, we first examined the impact of *PDCD10* silencing on cell proliferation, migration, and invasion in PA cell lines. As shown in Figure 2A, 2B, western blotting confirmed the knockdown efficiency of PDCD10-shRNA lentivirus. Besides, *PDCD10* silencing significantly increased the epithelial marker expression (E-cadherin) but reduced the mesenchymal marker expression (N-cadherin and VIM), demonstrating a significant inhibition of EMT process in PA cells (Figure 2A, 2B). The results of the CCK-8 assay showed significant suppression of cell proliferation after *PDCD10* silencing in Att-20 and TtT/GF cells (Figure 2C, 2D).

According to the scratch assay, the relative migration rate of Att-20 and TtT/GF cells in the *PDCD10*-shRNA group was 35.2±4.3% and 45.7±6.4%, respectively (Figure 2E, 2F). In addition, transwell invasion assays revealed that *PDCD10* silencing reduced numbers of invaded cells in Att-20 (65.2±11.6%) and TtT/GF (60.3±8.5%) cells (Figure 2G, 2H).

### Overexpression of PDCD10 promotes the proliferation, migration, invasion and EMT of PA cells

**Figure 3:**
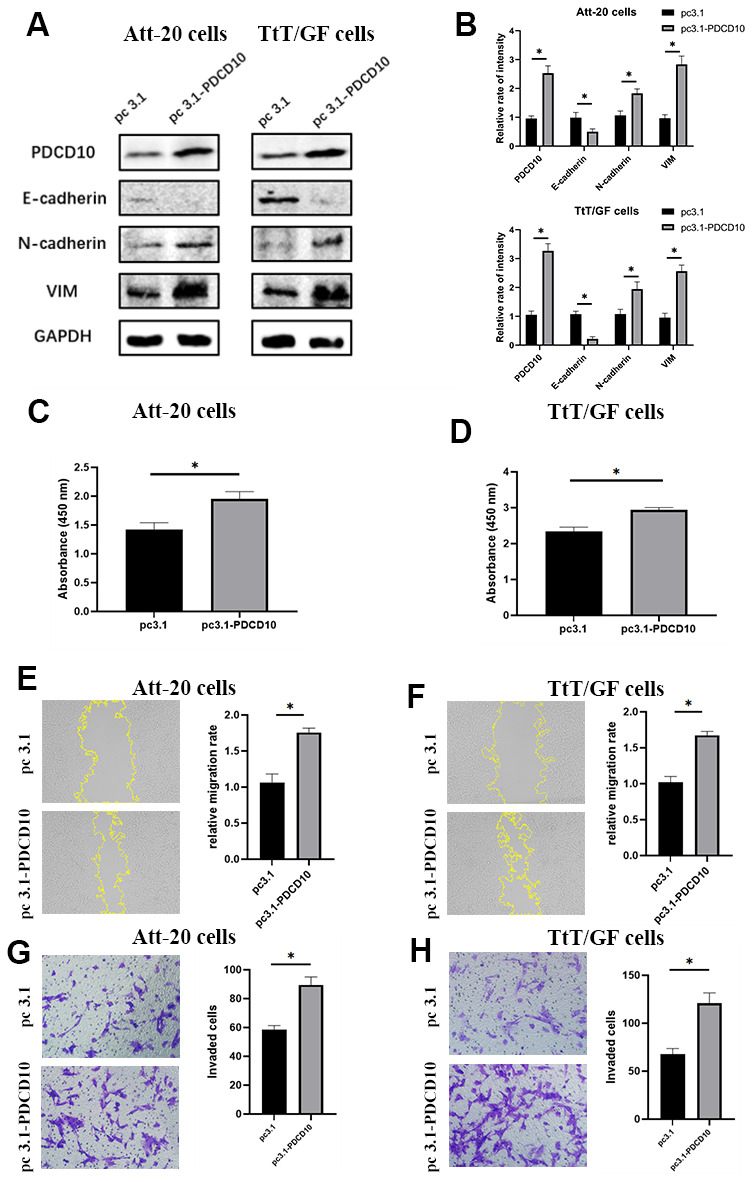
**Overexpression of *PDCD10* promotes the proliferation, migration, invasion and EMT of PA cells.** (**A**, **B**) Western blotting was performed to detect the impact of *PDCD10* overexpression on the expression levels of EMT markers in Att-20 cells and TtT/GF cells. Band intensities were quantified and normalized to GAPDH. (**C**, **D**) CCK-8 assay was used to assess cell proliferation potential after *PDCD10* overexpression in Att-20 and TtT/GF cells. (**E**, **F**) Scratch assay was employed to examine the relative migration rates of Att-20 and TtT/GF cells after *PDCD10* overexpression (magnification:100x). (**G**, **H**) Transwell invasion assay was used to detect the invasion potential of Att-20 and TtT/GF cells after *PDCD10* overexpression (magnification: 200x). * P < 0.05.

We further sought to verify the effect of PDCD10 in a gain-of-function setting, PDCD10-overexpressing PA cell lines were generated by transfection of pcDNA 3.1 plasmids containing the full-length coding sequence of murine *PDCD10*. The transfection efficiency was confirmed by western blotting (Figure 3A, 3B). Overexpression of PDCD10 reduced E-cadherin expression but increased the expression of N-cadherin and VIM, indicating a remarkable promotion of EMT process in PA cells (Figure 3A, 3B). Besides, up-regulation of PDCD10 remarkably promoted the capacities of proliferation (Figure 3C, 3D). The relative migration rate of Att-20 and TtT/GF cells in PDCD10 overexpression group was 168.7±7.4% and 163.8±6.3%, respectively (Figure 3E, 3F). Overexpression of PDCD10 increased numbers of invaded cells in Att-20 (56.2±13.2%) and TtT/GF (79.5±15.8%) cells (Figure 3G, 3H). Altogether, these results suggest that PDCD10 exerts a pro-oncogenic effect in PA cells.

### PDCD10 alters the protein expression level of CXCR2 and regulates the activation of downstream AKT/ERK signal pathways

**Figure 4:**
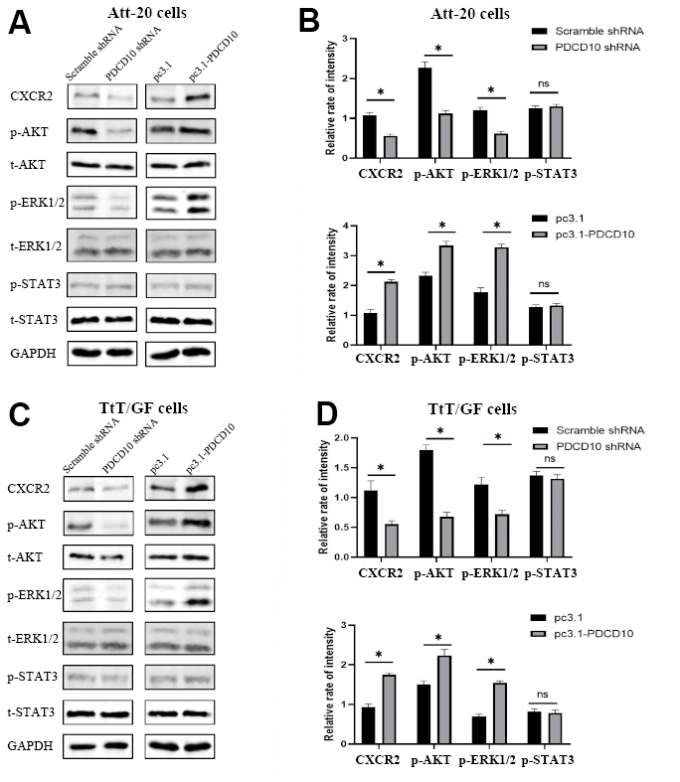
**PDCD10 alters the protein expression level of CXCR2 and regulates the activation of downstream AKT/ERK signal pathways.** (**A**, **B**) Western blotting was used to detect the expression level of CXCR2 and phosphorylation level of AKT, ERK1/2 and STAT3 in Att-20 cells after *PDCD10* silencing or overexpression. Band intensities were quantified and normalized to GAPDH. * P < 0.05. (**C**, **D**) In TtT/GF cells, western blotting was performed to examine the expression level of CXCR2 and phosphorylation level of AKT, ERK1/2 and STAT3 after *PDCD10* silencing or overexpression. Band intensities were quantified and normalized to GAPDH. * P < 0.05.

According to our previous study, CXCL2-CXCR2 signaling mediated by PDCD10 participates in the crosstalk between glioblastoma cells and microglia/macrophages and promotes tumor growth [25]. Therefore, we analyzed expression levels of CXCR2 in *PDCD10*-silencing and *PDCD10*-overexpressing PA cells. Figure 4 revealed that *PDCD10* silencing reduced the protein expression levels of CXCR2, while CXCR2 expression was elevated in the *PDCD10*-overexpression group of Att-20 and TtT/GF cells. Meanwhile, the activation state of downstream signaling pathways including AKT, ERK, and STAT3 was examined to further explore the underlying mechanism of PDCD10. As shown in Figure 4, *PDCD10* silencing significantly inhibited phosphorylation of AKT and ERK1/2 but not STAT3 in Att-20 and TtT/GF cells. In addition, compared with the control group, simultaneous activation of AKT and ERK1/2 was observed in *PDCD10*-overexpression Att-20 and TtT/GF cells.

### Activation of CXCR2 rescues the inactivation of AKT/ERK signaling and the tumor-suppressive effects induced by PDCD10 silencing

Based on the above results, we presumed that CXCR2 may participate in the regulation of PDCD10 in the aggressive behavior of PA cells. To verify the hypothesis, *PDCD10*-silencing cells were treated with recombinant mouse CXCL2 protein to induce the activation of CXCR2, followed by analyses of cellular proliferation, migration, and invasion.

**Figure 5:**
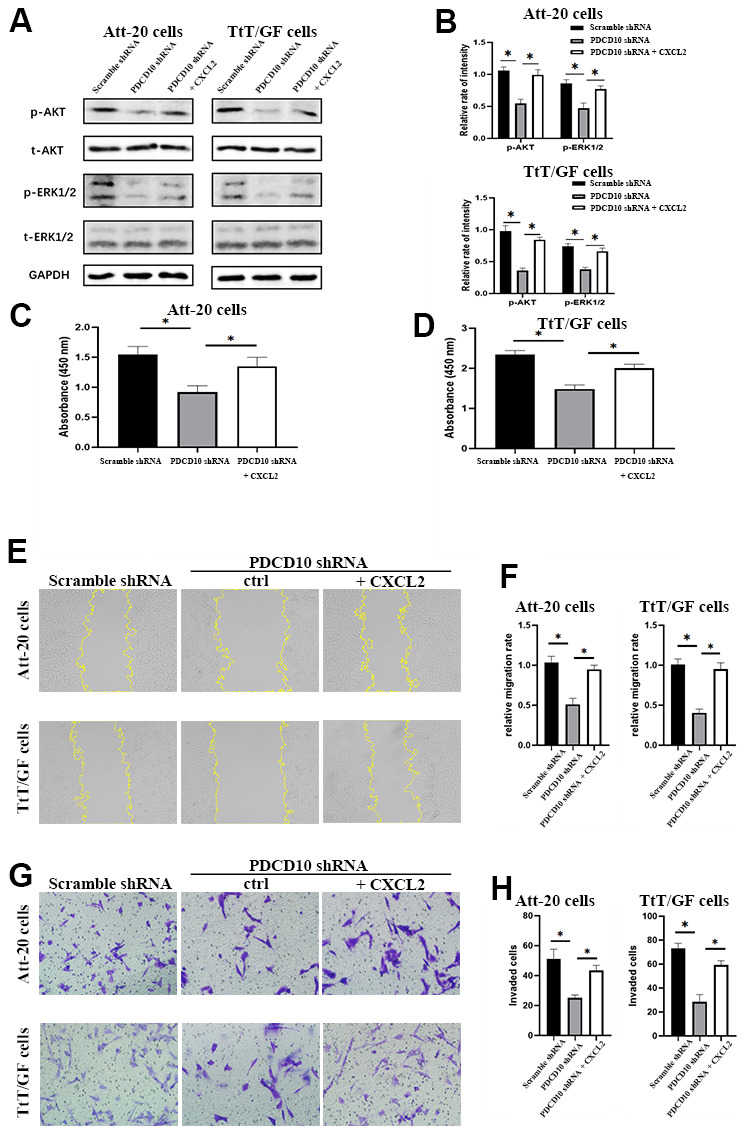
**Activation of CXCR2 rescues the inactivation of AKT/ERK signaling and the tumor-suppressive effects induced by PDCD10 silencing.** (**A**, **B**) Western blotting was performed to examine the phosphorylation levels of AKT and ERK1/2 after recombinant CXCL2 administration (200ng/ml) in Att-20 and TtT/GF cells with *PDCD10* silencing. (**C**, **D**) CCK-8 assay was used to evaluate cell proliferation potential after CXCL2 administration in Att-20 and TtT/GF cells with *PDCD10* silencing. (**E**, **F**) Relative migration rate of ATT-20 and TtT/GF cells with *PDCD10* silencing was analyzed by scratch assay after CXCL2 administration (magnification: 100x). (**G**, **H**) Transwell invasion assay was employed to assess the invasion capacity of Att-20 and TtT/GF cells with *PDCD10* silencing after CXCL2 administration (magnification: 200x). * P < 0.05.

After treatment with CXCL2, the phosphorylation levels of AKT and ERK1/2 were significantly increased (Figure 5A, 5B). Moreover, the inhibition of cellular proliferation induced by PDCD10 silencing was reverted via activation of CXCR2 by CXCL2 administration in Att-20 and TtT/GF cells (Figure 5C, 5D). CXCL2 treatment in *PDCD10*-silencing group also increased migration area (87.8±8.2% and 147.4±13.6%, respectively) and numbers of invaded cells (79.2±15.8% and 110±13.7%, respectively) in PA cell lines (Figure 5E–5H). These results suggest that PCDC10 and CXCL2 are part of the same signal cascade.

### PDCD10 silencing impairs the tumorigenesis and reduces CXCR2 expression of PA cells *in vivo*

**Figure 6:**
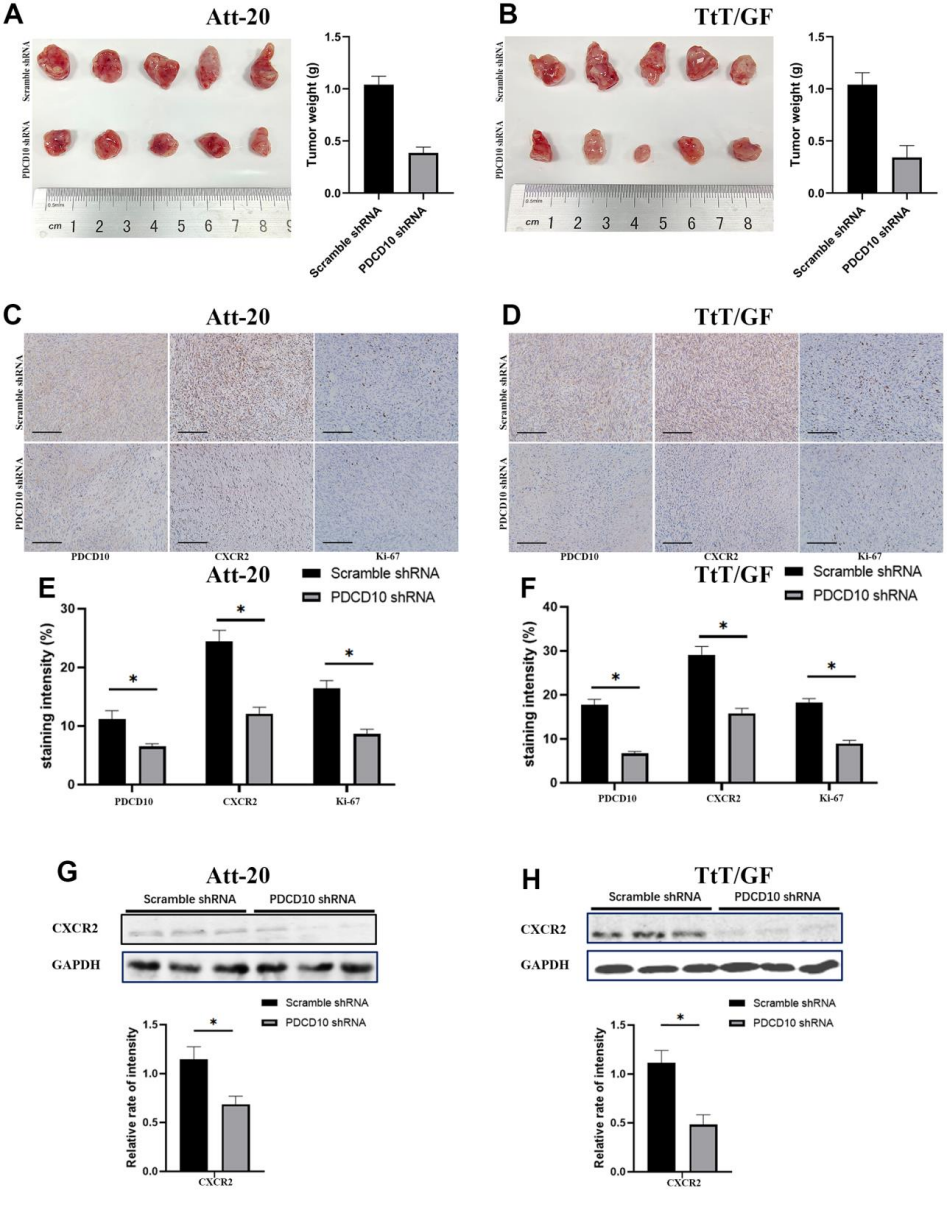
***PDCD10* silencing impairs the tumorigenesis and reduces CXCR2 expression of PA cells *in vivo*.** (**A**, **B**) Images for Att-20 and TtT/GF xenografts from nude mice (Left). Statistical analysis of xenograft tumor weights (Right). (**C**, **D**) Representative IHC images of Att-20 and TtT/GF xenograft tumor tissues for PDCD10, CXCR2 and Ki-67 staining (200x). Scale bars: 100μm. (**E**, **F**) Intensity of PDCD10, CXCR2 and Ki-67 staining were analyzed by IHC-Profiler. (**G**, **H**) Western blotting was performed to examine the expression of CXCR2 in Att-20 and TtT/GF xenograft tumor samples. Band intensities were quantified and normalized to GAPDH. * P < 0.05.

Finally, *in vivo* experiments were implemented to verify the effect of PDCD10 on tumorigenesis in PAs by establishing Att-20 and TtT/GF cell xenograft nude mice model with stable *PDCD10*-silencing. Consistent with the results from the *in vitro* experiments, *PDCD10* silencing significantly reduced the tumor sizes and weights of xenograft tumors (Att-20 cell line: 0.94±0.07g vs. 0.38±0.06g, and TtT/GF cell line:1.04±0.11g vs. 0.34g±0.12g) (Figures 6A, 5B). Besides, the Ki-67 staining, a proliferation indicator, was also decreased in *PDCD10*-silencing tumor samples according to IHC results (Figure 6C–6F). IHC staining and western blotting also further confirmed that *PDCD10* silencing decreased CXCR2 expression level *in vivo* (Figure 6C–6H). These data confirm that PDCD10 promotes tumor growth by regulating the expression of CXCR2 in PAs.

## DISCUSSION

As the second most common type of primary CNS tumors, high recurrence rates, and increased mortality as a consequence of aggressive PAs remain a great clinical challenge. The strong abilities of proliferation and infiltration/invasion of tumor cells result in large tumor size and severe destruction of adjacent normal structures, which is the major cause for incomplete removal of PAs and relapse [30]. However, the molecular and cellular mechanisms underlying the aggressive behaviors of PA cells need to be elucidated.

PDCD10 is an evolutionarily conserved protein and is widely distributed in a variety of tissues. According to a dataset from the GEO database, the expression levels of PDCD10 are increased in PA tissues compared to normal pituitaries (Figure 1A), suggesting that PDCD10 may exert a pro-tumorigenic effect in PAs.

Using loss-of-function as well as gain-of-function approaches, we demonstrate the pro-oncogenic effect of PDCD10 in Att-20 and TtT/GF cells: when expressed at high levels, PDCD10 enhanced the abilities of cellular proliferation, migration, invasion and epithelial to mesenchymal transition (EMT) in PAs. Some studies have indicated that PDCD10 plays an essential role in cell survival and migration. For example, Fidalgo et al. found PDCD10 can protect cells exposed to reactive oxygen species from death by mediating phosphorylation of the Ezrin/Radixin/Moesin proteins [31]. Proteomic analysis identified some proteins which regulate the cell cycle as interactors of PDCD10 [16, 32]. Under physiological conditions, PDCD10 is indispensable for neuronal migration during development [33]. In recent years, accumulating evidence has shown that dysregulation of PDCD10 was closely related to tumorigenesis and progression in diverse tumor types. Initially, PDCD10 was found to be associated with protein phosphatase-2A, a regulator of mitogenesis, and to enhance proliferation in malignant T cells [34]. Sun et al. reported that PDCD10 promotes the progression of hepatocellular carcinoma by interacting with PP2Ac to increase activation of YAP [21]. In ovarian cancer cells, PDCD10 upregulates Wnt/β-catenin signaling thereby augmenting migration of tumor cells [17]. Genome-wide analysis also showed that increased copy number and high expression level of PDCD10 are associated with tumor grade, nodal involvement and advanced FIGO stage in ovarian cancer [35]. In breast cancer, overexpression of PDCD10 induced the inactivation of the ROCK/Rho signal pathway to suppress cell adhesion and promote cell migration and invasion [20]. PDCD10 also participates in regulation of stemness of breast cancer cell [36]. Besides, PDCD10 was identified as the target of CircSMARCA5-miR-432 axis, and was found to promote proliferation, metastasis and glycolysis of prostate cancer cells [37]. The pro-tumorigenic effect of PDCD10 was also recently reported in lung cancer and bladder cancer [19, 38]. On the contrary, PDCD10 was reported as a tumor suppressor in glioblastoma cells [18]. These contradictory results hint that function of PDCD10 is tissue- and disease-specific in different tumors. As shown in our previous results, PDCD10 participates in the crosstalk between glioblastoma cells and microglia/macrophages and promotes tumor growth by CXCL2-CXCR2 signaling *in vivo* [25], which suggests that CXCR2 may play a critical role in aggressive behaviors mediated by PDCD10 in tumors. In this study, we report for the first time that PDCD10 can promote cellular proliferation, migration, and invasion of PAs by regulating the CXCR2-AKT/ERK signaling axis.

EMT is a cellular process that cells lose their epithelial features and acquire mesenchymal phenotype. It is well established that EMT is associated with tumor initiation, tumor cell migration, tumor stemness, metastasis, and treatment resistance in various cancer types [39]. Some studies have reported that EMT plays a key role in tumor progression of PAs [40, 41]. For example, Li et al. found that high CCNB1 expression resulted in cavernous sinus invasion of PAs through promoting EMT process [40]. PDCD10 was also reported to regulate EMT in hepatocellular carcinoma and ovarian cancer [17, 21]. Thus, it can be speculated that PDCD10 might contribute to tumor progression in PAs by inducing EMT. In our study, we observed that *PDCD10* silencing increased epithelial marker expression (E-cadherin), but reduced the mesenchymal marker (N-cadherin and VIM), while overexpression of *PDCD10* reduced E-cadherin expression but increased the expression of N-cadherin and VIM in PA cells. These results suggest that PDCD10 acts as regulators of EMT in PAs.

As a seven-transmembrane (7TM) protein, the C-terminus of CXCR2 is related to phosphorylation and internalization of the receptor, while the N-terminal dominant and extracellular loops determine the binding with ligands [42]. The ligands of CXCR2 include CXCL1, CXCL2, CXCL3, CXCL5, CXCL6, CXCL7, and CXCL8, among which CXCL1, CXCL2, CXCL3, CXCL,5, and CXCL7 bind CXCR2 only [43]. After binding of these cognate chemokine ligands to CXCR2 on the cell surface, the associated G-protein is activated and dissociates, which in turn leads to activation of multiple downstream signaling cascades including the phosphatidylinositol-3 kinase (PI3K)/Akt and RAS-Raf-MEK1/2-ERK1/2 signaling pathway [42].

To date, CXCR2 has been extensively studied in various types of neoplasms. In lung adenocarcinoma, up-regulation of CXCR2 in tumor cells promotes invasion and metastasis, which results in a poor prognosis [44]. In addition, high expression of CXCR2 has also been observed in PAs, medullary carcinomas of the thyroid, and pheochromocytomas [23]. CXCR2 was also reported to be associated with EMT and resistance of tumor cells to chemotherapy [45, 46]. Besides, CXCR2 was up-regulated in cancer stem cells (CSCs) and promotes the growth and migration of CSCs [47, 48]. In our study, we observed that PDCD10 silencing and overexpression significantly inhibited and increased protein expression level of CXCR2 together with phosphorylation levels of AKT and ERK1/2 in Att-20 and TtT/GF cells, respectively. In PAs, aberrant activation of AKT and ERK pathways was reported to lead to abnormal hormone production and neoplastic growth [49, 50]. Interestingly, activation of CXCR2 reversed the down-regulation of ERK1/2 and AKT phosphorylation level and suppression of proliferation, migration, and invasion induced by knockdown of PDCD10. Thus, we assumed that CXCR2 is involved in aggressive behaviors of tumor cells mediated by PDCD10 in PAs. Proteomic analysis revealed that PDCD10 may be involved in vesicle trafficking of membrane proteins [16, 32]. Consistent with this, several recent studies reported that PDCD10 was involved in regulation of exocytosis and AQP2 membrane targeting [51, 52]. Thus, we speculate that CXCR2 expressed as a membrane protein, maybe stabilized by PDCD10 and its interactors via regulating protein trafficking. However, the precise mechanism of how PDCD10 and CXCR2 cooperate in the cellular context requires further exploration in the future.

*In vivo*, PDCD10 silencing significantly reduced the sizes and weights of PA cell-derived tumors in xenograft models. In addition, the expression levels of Ki-67, which represents a level of tumor proliferation, together with CXCR2 were decreased in the PDCD10-shRNA group. These results further verify the pro-oncogenic effect of PDCD10 in PAs and imply the therapeutic potential of targeting PDCD10. Although temozolomide has been included in the guidelines of the Endocrine Society and European Society of Endocrinology, long-term use of it could result in an increase of toxicity including the risk of leukemia and bone marrow suppression [53–56]. Besides, the efficacy and safety of existing targeted therapies (e.g., RTK inhibitors) for PAs still need further investigation [57]. Thus, PDCD10 could be a new promising target for the design of drugs and treatment of PAs.
